# *In silico* evaluation of anti-colorectal cancer inhibitors by Resveratrol derivatives targeting Armadillo repeats domain of APC: molecular docking and molecular dynamics simulation

**DOI:** 10.3389/fonc.2024.1360745

**Published:** 2024-04-30

**Authors:** Shopnil Akash, Md. Rezaul Islam, Abdul Ali Bhuiyan, Mirza Nafeul Islam, Imren Bayıl, Rasha Mohammed Saleem, Ghadeer M. Albadrani, Muath Q. Al-Ghadi, Mohamed M. Abdel-Daim

**Affiliations:** ^1^ Department of Pharmacy, Faculty of Allied Health Sciences, Daffodil International University, Ashulia, Dhaka, Bangladesh; ^2^ Department of Pharmacy, Pabna University of Science and Technology, Pabna, Bangladesh; ^3^ Department of Pharmacy, University of Rajshahi, Rajshahi, Bangladesh; ^4^ Department of Bioinformatics and Computational Biology, Gaziantep University, Gaziantep, Türkiye; ^5^ Department of Laboratory Medicine, Faculty of Applied Medical Sciences, Al-Baha University, Al-Baha, Saudi Arabia; ^6^ Department of Biology, College of Science, Princess Nourah bint Abdulrahman University, Riyadh, Saudi Arabia; ^7^ Department of Zoology, College of Science, King Saud University, Riyadh, Saudi Arabia; ^8^ Department of Pharmaceutical Sciences, Pharmacy Program, Batterjee Medical College, Jeddah, Saudi Arabia; ^9^ Pharmacology Department, Faculty of Veterinary Medicine, Suez Canal University, Ismailia, Egypt

**Keywords:** drug design, resveratrol derivatives, colorectal cancer, molecular docking, molecular dynamics simulation

## Abstract

Colorectal cancer is the second leading cause of cancer-related deaths. In 2018, there were an estimated 1.8 million cases, and this number is expected to increase to 2.2 million by 2030. Despite its prevalence, the current therapeutic option has a lot of side effects and limitations. Therefore, this study was designed to employ a computational approach for the identification of anti-cancer inhibitors against colorectal cancer using Resveratrol derivatives. Initially, the pass prediction spectrum of 50 derivatives was conducted and selected top seven compounds based on the maximum pass prediction score. After that, a comprehensive analysis, including Lipinski Rule, pharmacokinetics, ADMET profile study, molecular orbitals analysis, molecular docking, molecular dynamic simulations, and MM-PBSA binding free energy calculations. The reported binding affinity ranges of Resveratrol derivatives from molecular docking were -6.1 kcal/mol to -7.9 kcal/mol against the targeted receptor of human armadillo repeats domain of adenomatous polyposis coli (APC) (PDB ID: 3NMW). Specifically, our findings reported that two compounds [(03) Resveratrol 3-beta-mono-D-glucoside, and (29) Resveratrol 3-Glucoside] displayed the highest level of effectiveness compared to all other derivatives (-7.7 kcal/mol and -7.9 kcal/mol), and favorable drug-likeness, and exceptional safety profiles. Importantly, almost all the molecules were reported as free from toxic effects. Subsequently, molecular dynamic simulations conducted over 100ns confirmed the stability of the top two ligand-protein complexes. These findings suggest that Resveratrol derivatives may be effective drug candidate to manage the colorectal cancer. However, further experimental research, such as *in vitro*/*in vivo* studies, is essential to validate these computational findings and confirm their practical value.

## 1 Introduction

Colorectal cancer (CRC), the third most common cancer globally, is the second leading cause of cancer-related death, with an estimated 1.8 million cases in 2018 and a expected increase to 2.2 million by 2030. CRC is currently the second most prevalent cancer among women (9.2%) and the third among men (10%) (1–3). CRC incidence is higher in developed countries (737,000 cases per year) compared to less developed regions (624,000 cases per year). This cancer originates from the abnormal proliferation of glandular epithelial cells in the colon, appendix, or rectum (4). CRCs can be caused by three mechanisms or a combination of them: microsatellite instability (MSI), chromosomal instability (CIN), or CpG island methylator phenotype (CIMP). Fearon suggests that the classical CIN pathway commences with mutations in the adenomatous polyposis coli (APC) gene. The APC gene functions as a traditional tumor-suppressive gene in both hereditary and sporadic colorectal malignancies (5, 6). Chemotherapy is still the most effective option for cancer treatment, but it’s becoming less effective as cancer cell lines develop resistance due to the undesirable side effects between cancerous and normal cells (7). To overcome this unwanted side effect, an alternative approach and novel therapeutic development are needed for the management, and treatment of CRC. However, the process of developing an effective and novel medication is challenging, lengthy, and costly, as well as requires substantial research resources. Besides, a large number of drugs fail during the development phase or pre-clinical or clinical trials due to undesirable side effects and toxicity, resulting in a huge amount of costs, and resources being wasted. Computational drug design approaches can reduce costs and resources in pharmaceutical development by minimizing early physiochemical and toxicity prediction studies. Thus, the current study incorporates the widely used computational approach (*in silico* study) to analyze the binding mode, dynamic simulation, and residual interaction with the target protein APC, aiming to determine the drug-like characteristics of Resveratrol derivatives effective in treating human CRC. In this investigation, we selected Armadillo repeats domain of Adenomatous polyposis coli (APC) as the targeted receptor. The guanine nucleotide exchange factor is activated by this substance to regulate cell-cell adhesion and migration. In CRCs, decreased APC leads to constitutive activation. This activation enhances the motility and angiogenesis of cancer cells in CRC. So, our primary target is to inhibit this Armadillo repeats domain of APC (8, 9). The Resveratrol derivatives have been chosen due to their wide range of therapeutic benefits, and pharmacological effects such as cardioprotective neuroprotective, antitumor, antidiabetic, antioxidants, anti-age effects, and glucose metabolism. It also reported strong pharmacological action against different types of cancer including colon cancer, and thyroid carcinoma as well as being capable of controlling oxidative stress, cell death, and inflammation (10–12).

## 2 Materials and methods

### 2.1 PASS prediction

In this study, we utilized pass prediction investigation to identify the possible pharmacological effectiveness of the Resveratrol derivatives using pass online website. This web-based program, has the capability to predict the bioactivity spectrum of a molecule by predicting the numerous probable pharmacological effects based on the molecule’s structure. The bioactivity prediction for compounds was conducted using the Pass online web server, accessible at https://www.way2drug.com/passonline/index.php. This platform enables the prediction of the bioactivity spectrum at different threshold values, specifically denoted as Pa (probable activity) and Pi (probable inactivity) (13, 14).

### 2.2 Preparation of ligand dataset

**Figure 1:**
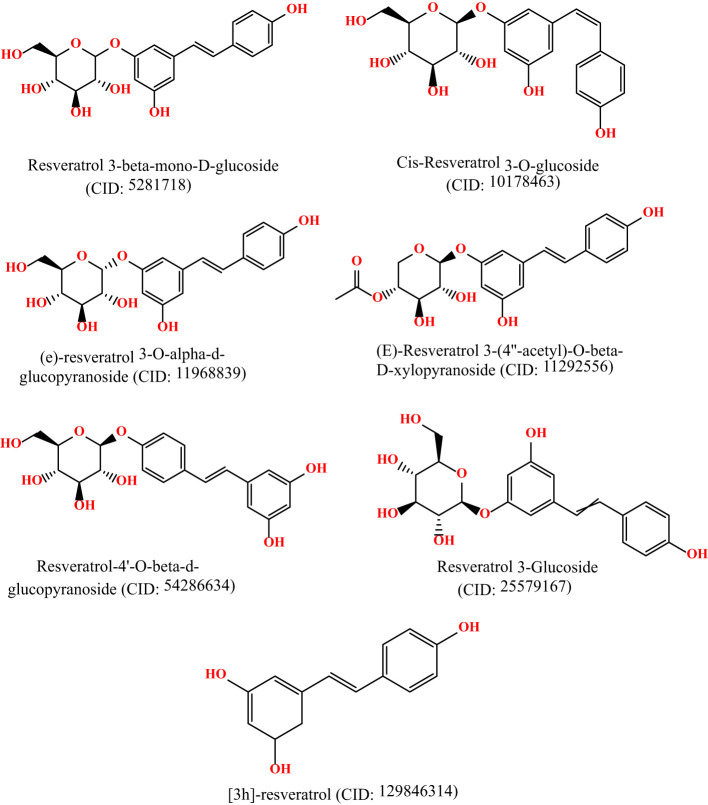
Molecular structure of selected ligands. Although, they have similar physicochemical properties. However, each of the ligands contains different structural shape, and functional unit.

The 3D chemical structures of Resveratrol analogs were initially acquired from the PubChem database (https://pubchem.ncbi.nlm.nih.gov/) in SDF format. Subsequently, the compounds were processed using BIOVIA Discovery Studio, incorporating hydrogens, and their structures were saved in PDB format. Following this, the ligands were optimized using Gaussian 09 software and the DFT/B3LYP 6- 311G approach before molecular docking (15, 16). These optimizations were conducted to ensure the ligands were prepared appropriately for subsequent molecular docking studies. The molecular structures are displayed in **Figure 1**.

### 2.3 Determination of ADMET profile

The study evaluated the potential of selected compounds as the mentioned drug candidates by evaluating their pharmacological profile using ADMET (Absorption, Distribution, Metabolism, Excretion, and Toxicity) analysis. Early-stage pharmacokinetic properties assessment is essential in computational drug design and development to identify the safety profile of newly develop drug candidates. Therefore, we studied the ADMET profile and drug-likeness properties of selected Resveratrol derivatives using the pkCSM (https://biosig.lab.uq.edu.au/pkcsm/prediction). This freely accessible web tools, pkCSM, utilization a state-of-the-art approach based on graph signaling to assess pharmacokinetic profiles (17).

### 2.4 Protein preparation and molecular docking

**Figure 2:**
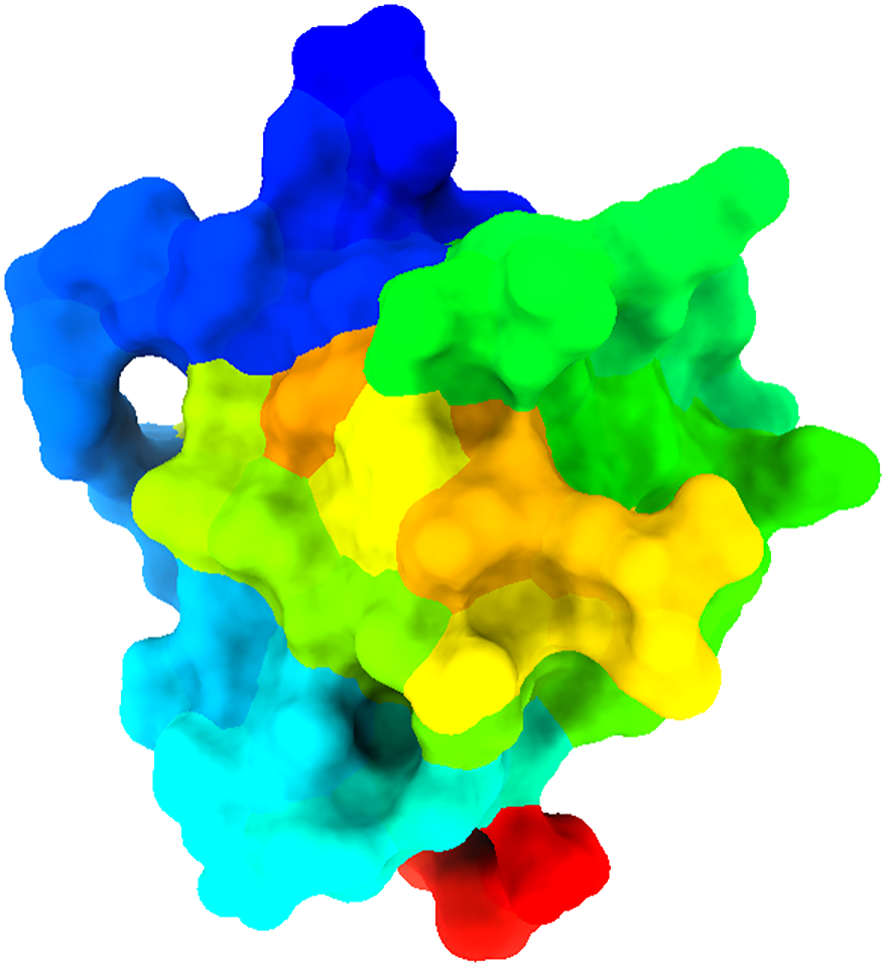
Displayed three-dimensional structure of selected target proteins.

The 3D Crystal structure of armadillo repeats domain of APC (Adenomatous polyposis coli) tumor suppressor protein (PDB ID 3NMW) was retrieved from the RCSB protein Data Bank (https://www.rcsb.org/). The protein crystal structure was resolved by the X-ray diffraction method with a resolution of 1.60 Å. The PDB protein structure was refined/purified by removing water molecules, unwanted heteroatoms, and ligands that were already attached with protein that may interfere with the desired ligand-protein binding, and by keeping the desired chain fold in BIOVIA Discovery Studio Visualizer. Then, the structure (**Figure 2**) was prepared through Biovia discovery studio. After preparation of the protein, the molecular docking work was performed to predict the trajectory of the binding potential of a protein-ligand complex using PyRx virtual screening tool AutoDock Vina. This study employed the default configuration parameters of the AutoDock Vina Wizard, a virtual screening tool developed by PyRx. Before conducting docking, the set of grid parameters was selected based on receptor active sites to conduct the molecular docking work. For further evaluation, the result displaying the highest binding affinity (kcal/mol) with a negative sign was elected. Finally, we visualized the protein-ligand interaction complex to identify the strong binding pose of active site residues with the lead compounds and their bond distance using the BIOVIA Discovery Studio Visualizer.

### 2.5 Molecular dynamics simulation

Molecular Dynamics (MD) Simulations were carried out to investigate the validity of the applied molecular docking studies. As known that MD simulation is a principal tool for proofing the stability of protein-ligand MD simulation provides detailed information on the fluctuations and conformational changes of proteins-ligand complex towards its stable conformation (18). Thus, in the present study, the MD simulations of the targeted human armadillo domain of APC (PDB ID: 3NMW) protein with selected ligands complexes have been conducted to establish the binding accuracy of 03, 29, and standard capecitabine to the targeted protein (PDB ID: 3NMW). The computations were performed for 100 nanoseconds in a periodic water box utilizing the Gromacs version 2020 software package and the CHARMM36 force field (19). Using the CHARMM-GUI web-based graphical interface, the simulation system was configured and the force fields for both the ligands and the protein were generated (20). The complexes were positioned within a rectangular box that had a buffer distance of 10 in each of the cardinal directions. The box was subsequently dissolved in water molecules containing TIP3P (21, 22). In order to maintain system neutrality, sodium and chloride ions were put into the system, followed by the minimization of energy using the steepest descent method. The equilibration process was carried out on all of the full systems at a temperature of 310 K for a total of 5,000 steps, equivalent to 10 picoseconds (PS). An isothermal-isochoric ensemble NVT (constant number of particles, volume, and temperature) and an isothermal-isochoric ensemble NPT (constant number of particles, pressure, and temperature) were used to equilibrate each system (22, 23). To restrict hydrogen, the Lñncs algorithm was employed; hence, the time step was established at 2 fs.

We conducted a comprehensive assessment of all van der Waals (vdw) forces using a switching system that varied from 12 to 14 Ã, with a cutoff value of 14. The long-term electrostatic connections have been determined using the particle mesh Ewald (PME) method with a maximum grid spacing of 1.2. Instead of utilizing a multiple-time-stepping approach, PME processes have been carried out at every step. The temperature kept constant at 310 K. The barostat was set to regulate system size fluctuations to a concentrated level of 1 bar (24, 25). The time step for integration was 2 femtoseconds. Upon completion of the 100 ns molecular dynamics simulation, the simulation outputs were initially re-centered. Subsequently, the trajectory files were analyzed using Gromacs and VMD built-in tools to investigate the dynamic conformational changes and interactions within the complexes over time. Analyzed simulated trajectories to calculate Root Mean Square Deviation (RMSD), Residue Root Mean Square Fluctuation (RMSF), radius of gyration growth, number of H-bonds, Principal Component Analysis (PCA), and Dynamics Cross-Correlation Matrices (DCCM).

### 2.6 Binding free energy calculation using MM-PBSA

In this study, we utilized the MM-PBSA approach to compute the free interaction energy between molecules and the targeted protein (PDB ID: 3NMW). Estimation of binding free energy (ΔG) was performed using Equation (1) with the script MMPBSA.py from the AMBER package (26).


(1) $$\text{ΔG}={\text{G}}_{\text{complex}}-{\text{G}}_{\text{receptor}}-{\text{G}}_{\text{ligand}}$$

G-complex is the free energy of the complex; G-receptor is the free energy of the receptor; G-ligand is the free energy of the ligand (27).

## 3 Results analysis

### 3.1 PASS prediction

The pass prediction properties was utilized to identify potential therapeutic properties of the compounds. The PASS prediction value evaluates a compound’s similarity to known physiologically relevant structures, allowing scientists to predict a molecule’s activity. This strategy can be employed from the beginning of the study to develop a new medication (28, 29). The previously obtained structures of the described compounds were submitted as a mol form to the PASS online program, and the potential therapeutic activities were investigated. These filters are based on the probability of potential activity (Pa) as well as inactivity (Pi) (13). PASS algorithms and filters can help scientists efficiently screen through thousands of lead candidates, saving time and money by focusing on the most promising ones. Which substantially reduces the time it takes to discover novel medications and increases their chances of effectiveness in treating a wide range of diseases.

Pa and Pi values can range from 0.00 to 1.0, and notably, Pa+Pi≠1, demonstrating that a molecule cannot be simultaneously active and inactive at the same time (30). In this investigation, total 50 Resveratrol derivatives were taken, and analysis pass prediction (**Supplementary Table 1**). Regarding the data, our reported compounds had the most effective antineoplastic activity, exhibiting the Pa value above 0.775+ by the majority of the compounds and considering Ligand no 03, 11, 17, 24, 28, 29, and 39 demonstrated the highest Pa score (Pa > 0.791, Pa > 0.791, Pa > 0.791, Pa > 0.833, Pa > 0.791, Pa > 0.791, and Pa > 0.775) respectively. Where the range of Pa values for antibacterial, antifungal, and antiparasitic was between 0.548-0.301, 0.676-0.421, and 0.480-0.363 correspondingly. These considerable Pa values along with favorable Pi values underline their suitability as antineoplastic agents, and based on these noticeable features, and top seven compounds is finally selected (**Table 1**).

**Table 1:** Pass prediction data of Resveratrol derivatives.

No	Name of the derivatives	PubChem ID	Antibacterial		Antifungal		Antineoplastic		Antiparasitic	
			Pa	Pi	Pa	Pi	Pa	Pi	Pa	Pi
03	Resveratrol 3-beta-mono-D-glucoside	5281718	0.548	0.012	0.676	0.011	0.791	0.013	0.480	0.018
11	Cis-Resveratrol 3-O-glucoside	10178463	0.548	0.012	0.676	0.011	0.791	0.013	0.480	0.018
17	(E)-resveratrol 3-O-alpha-d-glucopyranoside	11968839	0.548	0.012	0.676	0.011	0.791	0.013	0.480	0.018
24	(E)-Resveratrol 3-(4’’-acetyl)-O-beta-D-xylopyranoside	11292556	0.547	0.012	0.664	0.012	0.833	0,008	0.380	0.035
28	Resveratrol-4’-O-beta-d-glucopyranoside	54286634	0.548	0.012	0.676	0.011	0.791	0.013	0.480	0.018
29	Resveratrol 3-Glucoside	25579167	0.548	0.012	0.676	0.011	0.791	0.013	0.480	0.018
39	[3h]-resveratrol	129846314	0.301	0.060	0.421	0.046	0.775	0.015	0.363	0.039

### 3.2 ADMET profile investigation

The pharmacokinetic features of the compounds provided better understood through the application of in silico ADMET analysis, which is valuable for evaluating their therapeutic potential (**Table 2**). One of the essential factors influencing the distribution and absorption of drugs is their water solubility, indicated by the parameter log S. The water solubility values of our compounds range from -2.575 to -2.892 to -3. 401.The following are the ordinary solubility ranges: insoluble<10, weakly soluble<6, moderately soluble< 4, soluble< 2, extremely soluble< 0, and very soluble > 0 (31–34). The stated water solubility values of our compounds vary fall within these ranges as well. According to these measurements, the solubility of our compounds ranges from moderate to poor.

**Table 2:** Summary of calculation of ADME results for selected derivatives.

No	Absorption		Distribution		Metabolism		Excretion		Toxicity		
	Water solubility Log S	Human Intestinal Absorption (%)	VDss (human)	BBB Permeability	CYP3A4 Inhibitor	CYP3A4 Substrate	Total Clearance (ml/min/kg)	Renal OCT2 substrate	Max. tolerated the dose (Log mg/kg/day)	Skin Sensitization	Hepatotoxicity
03	-2.575	51.086	0.125	-1.029	No	No	0.057	No	0.569	No	No
11	-2.892	78.122	0.011	-1.692	No	No	-32.29	No	0.438	No	No
17	-2.575	51.086	0.125	-1.029	No	No	0.057	No	0.569	No	No
24	-3.401	67.277	0.15	-1.056	No	No	0.257	No	-0.305	No	No
28	-2.892	77.387	0.011	-1.382	No	No	-31.43	No	0.438	No	No
29	-2.892	78.122	0.011	-1.692	No	No	-32.29	No	0.438	No	No
39	-2.028	89.45	0.601	0.043	No	No	0.151	No	-0.315	No	No

The intestinal absorption fraction of a drug is a key indicator of its absorption rate in the gastrointestinal tract. The absorption rates vary among the compounds, ranging from 51.086% to 89.45% where the compound 11, 28, 29, and 39 has reported optimum GI absorption. Additionally, our molecules exhibit volume of distribution (VDss) values such as 0.125, 0.011, and 0.15, reflecting how a medicine is distributed through the circulatory system.

To reach the central nervous system of any drugs or bioactive compounds require high blood-brain barrier (BBB) permeability capacity. Mentioned ligands has demonstrate a range of permeability values, from -1.029 to -1.692 and -1.056 which means they are almost unable to reach BBB, and the compounds has a chance to reach BBB since it shows 0.043. Furthermore, we assessed the potential impact of the compounds on CYP3A4, an essential enzyme in drug metabolism. The compounds have reported that neither act as substrates nor inhibitors of CYP3A4.

We also evaluate the renal OCT2 substrates and total clearance, which has been used to described the drug removal routes. Our finding has been documented that none of the compounds renal OCT2 substrate activity. In addition, total clearance values such as 0.057 and 0.257 are reported which means minimum 0.057 ml/min/kg excrete and maximum 0.257 ml/min/kg excrete from the body.

In the assessment of the toxicity, we considered skin sensitivity, hepatotoxicity, and maximum tolerated dosage. The compounds have totally free from hepatotoxicity and skin sensitization characteristics, and the minimum, and maximum tolerated dosage was documented 0.305 log mg/kg/day, and maximum 0.569 log mg/kg/day.

Finally, it is concluded that safety profiles of these mentioned derivatives are significant, confirming their potential as promising therapeutic candidates.

### 3.3 Molecular orbitals and chemical reactivity indicators

The compounds’ optimized geometry, highest occupied molecular orbital (HOMO) surfaces, lowest unoccupied molecular orbital (LUMO) surfaces, and molecular electrostatic potential (MEP) surfaces were calculated in the gas phase using the GaussView 5.0 molecular visualization program and Gaussian 09. The calculations were performed without symmetry restrictions, employing the DFT/B3LYP hybrid functional and 6-31G basis set (35–38). The representation of chemical reactivity and kinetic stability in a molecule is described by its frontier orbitals, which are essential for determining the bioactivity of compounds. There are two types of frontier orbitals in molecules: HOMO and LUMO. The shift of the electron from the lowest to the highest energy state is mainly measured for by the excitement of one electron from HOMO to LUMO (39, 40). Therefore, the transfer of electrons from the HOMO to the excited LUMO results in a substantial increase in energy. The kinetic stability of a molecule is a linear relationship between the HOMO-LUMO energy gap, which is described as increasing the HOMO-LUMO energy gap, simultaneously growing the chemical reactivity and kinetic stability (41, 42). **Table 3** presents the calculated values of molecular orbital energies, which include the two widely recognized chemical parameters: energy gap, chemical potential, electronegativity, hardness, and softness. The compound 11, as described, exhibited the greatest HOMO-LUMO energy gap (4.54648 eV), indicating a more stable structure. Furthermore, the derivatives exhibiting the highest softness value were calculated to be 0.56071 in Ligand 39. This indicates that the compound has the potential to dissolve at a faster rate, as shown in **Table 3**. At the same time, Ligand 11 exhibits a maximum hardness of approximately 2.7324. This hardness value suggests that it may have taken longer to break down after it entered the physiological system.

**Table 3:** Presents data on the chemical reactivity and molecular characteristics.

Name	HOMO	LUMO	Energy GAP	Hardness	Softness
03	-5.3290798	-1.2789356	4.05014	2.0250	0.49381
11	-5.700515	-1.154035	4.54648	2.7324	0.36597
17	-5.5337103	-1.4971712	4.03653	2.0182	0.49547
24	-5.4999673	-1.4647894	4.03517	2.0175	0.49564
28	-5.3655430	-1.2955345	4.07000	2.0300	0.49139
29	-5.16200184	-1.0846462	4.07735	2.0386	0.49051
39	-5.10975596	-1.542886159	3.566869	1.7834	0.56071

### 3.4 Molecular electrostatic potential

**Figure 3:**
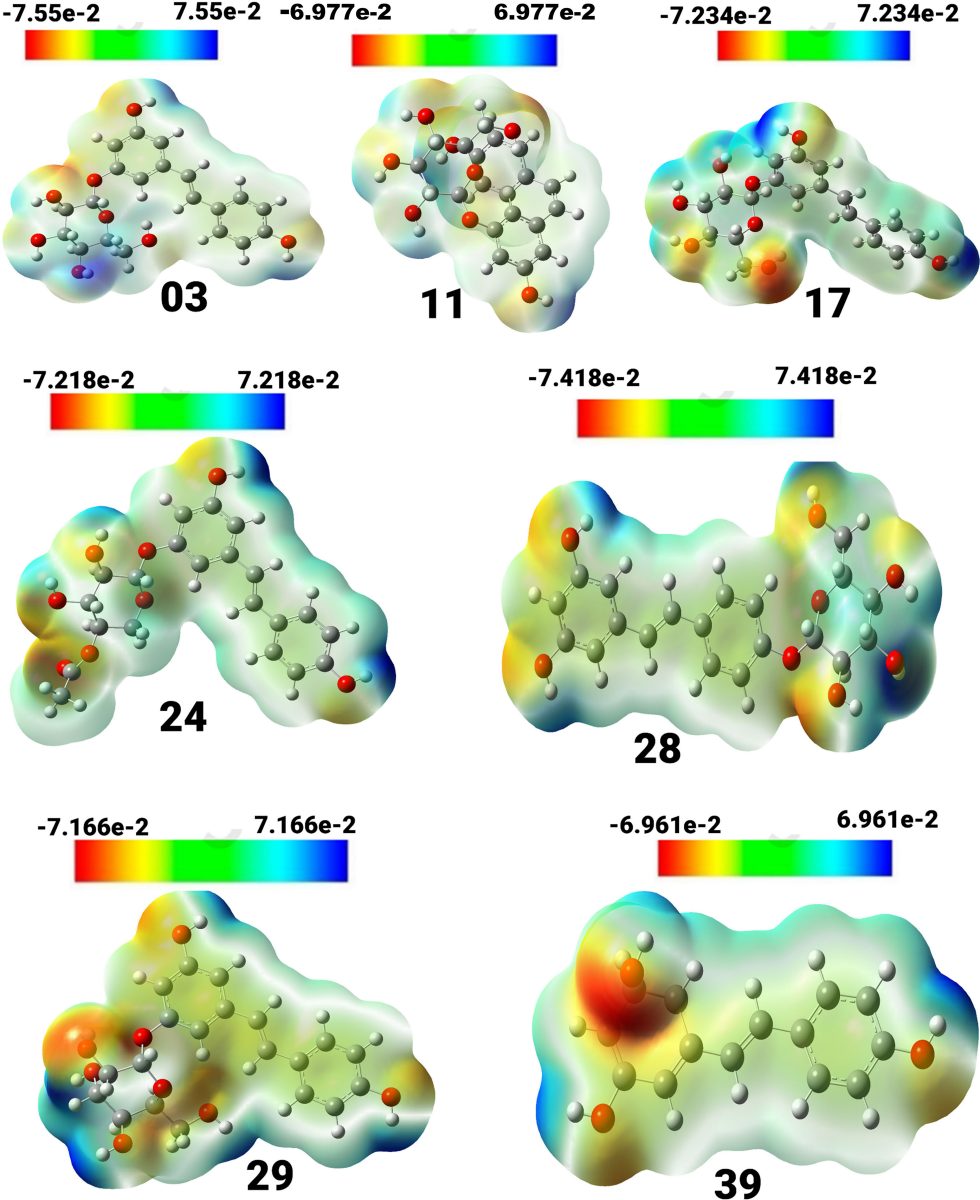
View of the molecular electrostatic potential surface of the ground state of the optimized structure of compounds, obtained by using the DFT/B3LYP/6-31G method.

Molecular Electrostatic Potential (MEP) provides extensive insights into studies associated with the chemical reactivity or biological activity of a product. The spatial arrangement and magnitude of electrostatic potential significantly influence the fundamental processes of chemical reactions, affecting the interaction of electrophilic or nucleophilic agents (43, 44). The investigation involved simulating the electrostatic potential map of the targeted compounds using the B3LYP method with a 6-31G basis set. The structure was then optimized, resulting in **Figure 3**. Color variations are used to visually represent the molecule’s structure, size, and regions of positive, negative, and neutral electrostatic probability. Additionally, it is a notable method for investigating the correlation between physicochemical properties and the structure of a specific substance. The decreasing potential of the attacking area occurs in the sequence of blue, red, and white. The white section represents neutral areas, while the red color indicates high electron saturation, indicating potential electrophile attack. The blue color indicates the lowest electron density surface, making it susceptible to nucleophilic attack.

Compound (3) displayed a spectrum of units ranging from -7.55e-2 to 7.55e-2 units, with colors ordered in the sequence of red, yellow, green, and blue. Compound (11) exhibited a unit range of -6.977e-2 to 6.977, while compound (17) showed a range of -7.234 to 7.234. Similarly, compound (24) exhibited a range of units between -7.218 and 7.218, and compound (28) displayed a range between -7.418 and 7.418. Compound (29) showed a spectrum of values ranging from -7.166 to 7.166, whereas compound (39) had a range between -6.961 and 6.961. The positive charges in compounds 17, 24, 39, 28, and 03 are mainly found on the hydrogen atoms of the hydroxyl groups connected to the aromatic rings. Conversely, the negative charges are predominantly concentrated on the oxygen atoms of the hydroxyl groups attached to the aromatic rings and are visually represented in red.

### 3.5 Molecular docking analysis against breast, and colorectal cancer targeted proteins

The molecular docking approach is a significant aspect of structural biology that is primarily employed for Computer-aided drug design (CADD). This is a technique for predicting the optimal binding mode of drug molecules to a specific receptor macromolecule (45, 46). In order to investigate the binding affinity between selected molecules and the target protein, we have therefore integrated molecular docking analysis in our study. The PyRx AutoDock software was used to assess possible binding energy and interaction with the active regions of CRC targeting protein.

Resveratrol derivatives (ligand numbers 11, 17, 24, 28, and 39) showed considerable binding affinity against the human armadillo repeats domain of APC (PDB ID: 3NMW) with predicted binding scores of -6.3 kcal/mol, -6.9 kcal/mol, -7.3 kcal/mol, -7.0 kcal/mol, and -6.1 kcal/mol, respectively (**Table 4**). In addition, the findings indicated that two compounds (ligand no: 03 and 29) displayed the maximum effectiveness compared to all other derivatives, with the maximum binding affinity values of -7.7 kcal/mol and -7.9 kcal/mol, respectively. Finally, it might be stated that these mentioned derivatives of Resveratrol perform a stronger binding affinity than the standard FDA-approved drug capecitabine, which ultimately suggested as possible therapeutic options for CRC therapy. However, further experimental research is required to validate these findings.

**Table 4:** Binding affinities of docked ligand calculated against targeted proteins.

SL. No	Armadillo repeats domain of APC (PDB ID: 3NMW)
	Kcal/mol
03	-7.7
11	-6.3
17	-6.9
24	-7.3
28	-7.0
29	-7.9
39	-6.1
Standard Capecitabine	-5.9

### 3.6 Molecular docking and interaction analysis

**Figure 4:**
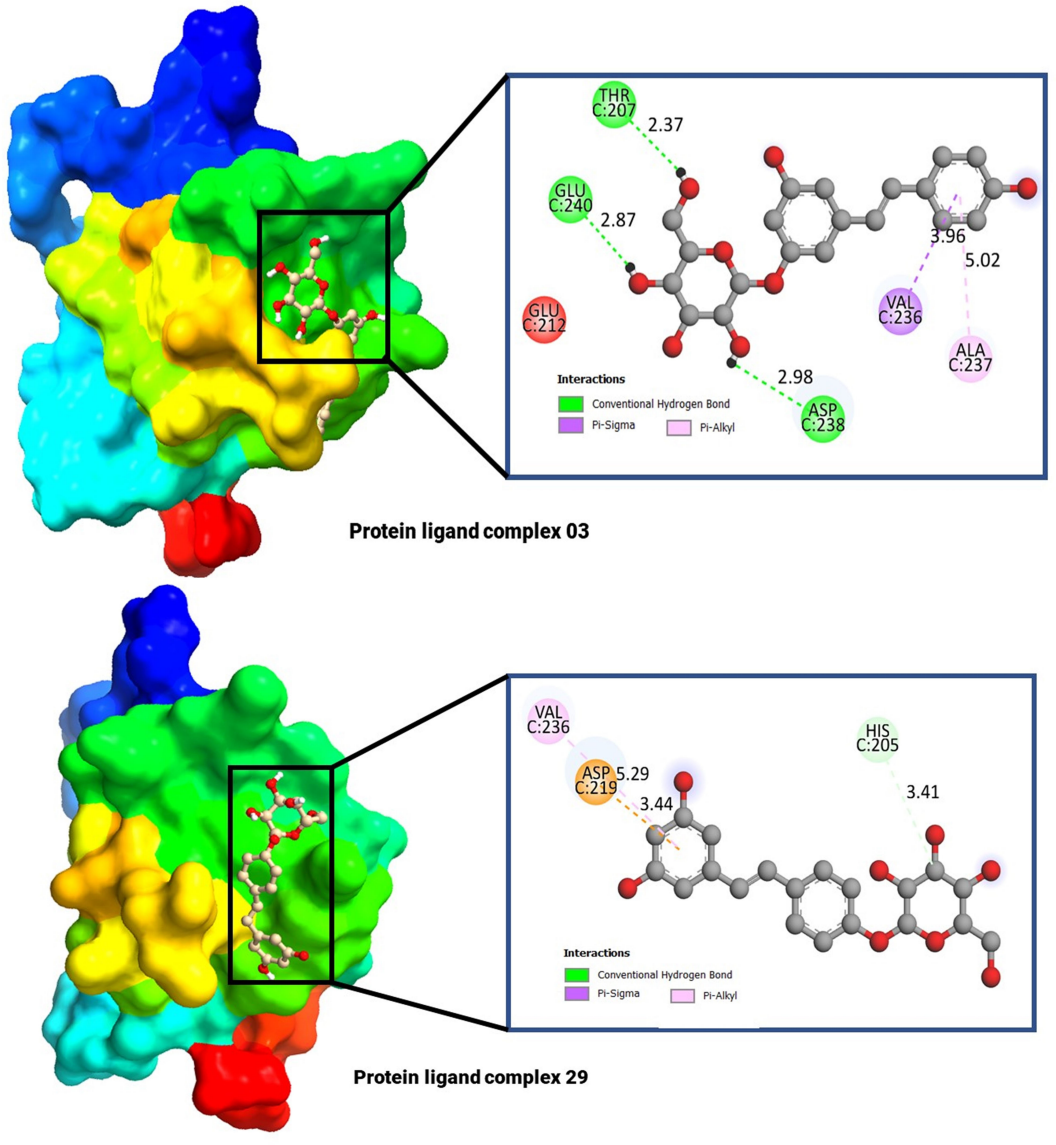
Molecular docking pocket, and active site analysis.

The molecular docking poses and active amino acid residues are schematically displayed in **Figure 4**. The Chimera application and Biovia Discovery Studio 2021 used to create this artwork. The molecular docking poses provided insights into the interaction between the drug and each target protein, highlighting the bonding strength of the reported ligand with these proteins (47, 48). It has been seen that the complex 03 has formed total five bonds where three are hydrogen bonds (THR C: 240, and ASP C: 238), and the complex 29 has documented total including HIS C: 205 (Hydrogen bonds), and the others two are ASP C: 219, and VAL C: 236.

### 3.7 Molecular dynamic simulation result analysis

#### 3.7.1 Root mean square deviation analysis (RMSD)

**Figure 5:**
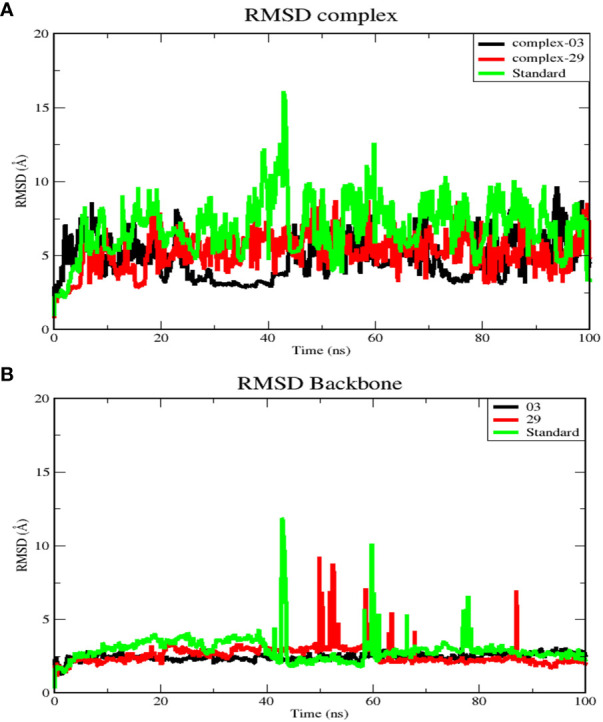
**(A)** RMSD for complex systems of 03, 29, and standard protein. **(B)** RMSD for backbone atoms of 03, 29, and standard protein.

RMSD analysis reveals deviations observed during the simulation progression. Furthermore, RMSD quantifies the stability of the structure, with lower RMSD values indicating greater stability (49). The RMSD calculations were performed on the protein backbones and complexes during a 100-nanosecond MD for each protein-ligand complex. This analysis provided valuable insights into the conformational changes occurring during protein-ligand interactions. The results are illustrated in **Figure 5**.

**Figure 5A** displays the average RMSD values for complex-03, complex-29, and the standard, which are 4.97, 5.4, and 7.18 Å, respectively. Initially, the RMSD of all complexes increased until 20 ns, after which each complex exhibited distinct RMSD patterns during the simulation. Between 25 and 45 ns, complex-03 experienced a dramatic decrease in its RMSD value. Conversely, both complex-29 and the standard complex exhibited an increase in their RMSD values, with the RMSD value of the standard complex reaching its peak. This increase indicates that the ligand’s affinity for the standard complex is relatively low. Following this time frame, every complex formation displayed fluctuations until the conclusion of the simulation.

Additionally, **Figure 5B** presents RMSD analysis of the backbone atoms for the three complexes during a 100 ns simulation, revealing different conformational states the backbone of the protein (PDB ID: 3NMW). The RMSD plots of the backbones of the complex 03, 29, and standard have mean values of 2.5, 2.6, and 2.1 Å, respectively. Among the three trajectories, the standard protein backbone exhibits the largest variation but has the lowest RMSD value. In contrast to the 29 and standard structures, the 03-protein backbone remained stable throughout the simulation.

#### 3.7.2 Root mean square fluctuation (RMSF) analysis

**Figure 6:**
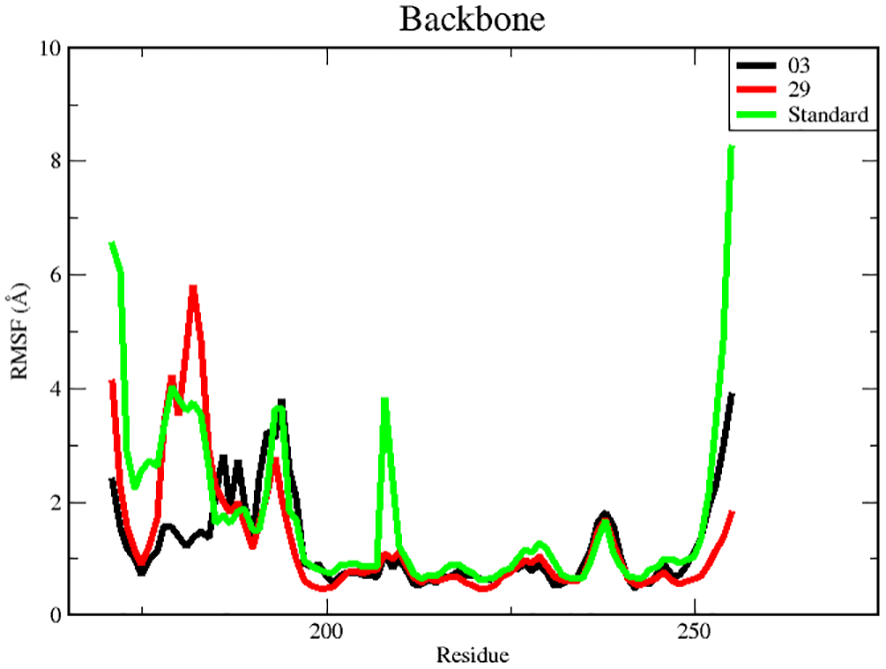
RMSF for backbone atoms of protein.

RMSF is a useful tool for evaluating the residual flexibility of a protein’s backbone, which is a essential factor in MD modeling. The protein residues are essential for attaining a stable conformation in a protein-ligand complex, and this stability can be assessed using the RMSF measure. A high RMSF number suggests increased flexibility, whereas lower RMSF values imply a more stable zone. Hence, residues or groups exhibiting RMSF values signify increased flexibility, suggesting a larger probability of interacting with ligand molecules. On the other hand, decreased RMSF fluctuations are linked to reduced flexibility, leading to a decrease in interaction capability (50, 51). In this study, we calculated RMSF values to investigate the effect of ligands on the residual flexibility of the protein backbone, as shown in **Figure 6**.

There were three different average backbone RMSF values: 1.22 Å for compound 03, 1.34 Å for compound 29, and 1.35 Å for the standard (**Table 5**). The RMSF graph for compound 03 exhibited strong peak fluctuations at the backbone residue positions of SER171-HSD170, LEU185-ALA186, SER192-VAL196, VAL236-GLU240, and VAL252-ASP255, as illustrated in **Figure 6**. For compound 29, an RMSF graph showed peak fluctuations at the backbone residue positions between PRO178-GLU183, SER192-GLY194, VAL236-GLY239, and ASN253-ASP255. In the case of the standard compound, an RMSF graph revealed peak fluctuations at the backbone residue positions between PRO178-GLU183, MET208-ASP210, THR227-GLU230, ALA237-GLY239, and ASN253-ASP255. These findings indicate that, in **Figure 6**, the protein backbone for compound 03 exhibits the lowest RMSF value, whereas compounds 29 and the standard compound have higher RMSF values.

**Table 5:** Average backbone of RMSF values.

No	RMSF
Ligand-03	1.22 Å
Ligand-03	1.34 Å
Standard	1.35 Å

#### 3.7.3 Radius of Gyration (Rg) analysis

**Figure 7:**
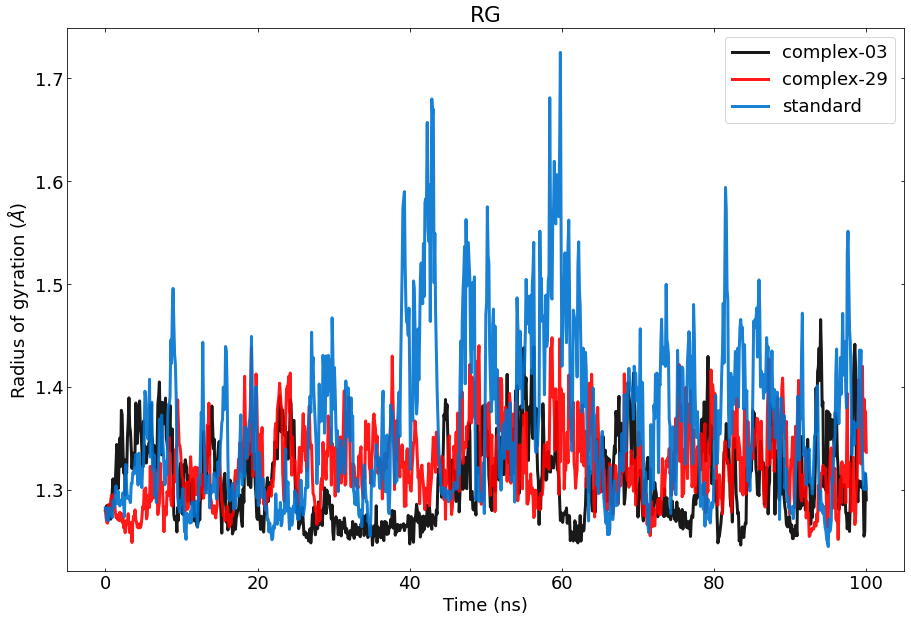
Represents the ROG values of the protein–ligand complexes to the protein backbone for 100 ns. RoG of 03, 29, and standard are shown in black, red, and blue respectively.

The Rg of a protein-ligand interaction complex refers to the distribution of its atoms about its axis. Calculating the Rg is a vital parameter for evaluating the structural dynamics of a macromolecule, as it provides insights into the changes in the overall compactness of the complex during the simulation. **Figure 7** depicts the the stability of therapeutic candidate compounds, specifically complex 03 (represented by the color black), complex 29 (represented by the color red), and the control standard (represented by the color blue), in their interaction with the target protein. This investigation focused on measuring the Rg values over the simulation period of 100 ns. Overall, the variations in Rg values among all complexes exhibited clear and unique patterns. According to the data, complex-03 exhibited the lowest Rg value, suggesting that it is more condensed compared to the other complexes. A decrease in density might result from a modification in the way the protein and the ligand interact with each other.

#### 3.7.4 Hydrogen bond analysis

**Figure 8:**
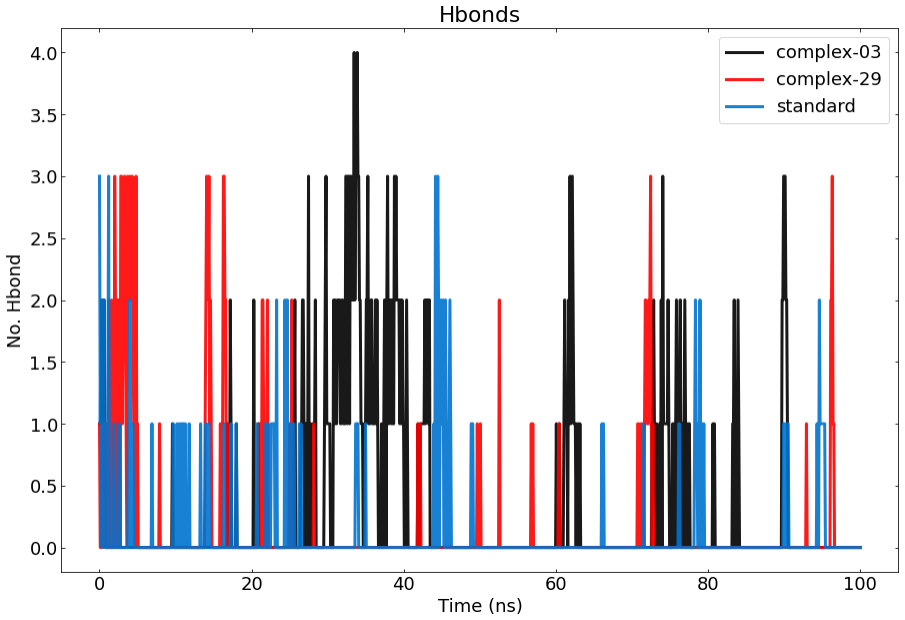
Represents the number of hydrogen bonds responsible for the stability of the complexes (03, 29, and standard) throughout the 100 ns.

The results of calculating and graphing the number of hydrogen bonds established by the ligand molecules (ligand-03, ligand-29, and the standard) with the proteins are shown in **Figure 8**. The presence of intermolecular hydrogen bonding between the protein and the ligand is essential for stabilizing protein-ligand complexes. The stability of the hydrogen bond network formed by the ligands (ligand-03, ligand-29, and the standard) has been assessed throughout the 100-nanosecond simulation period. Hydrogen bond conditions were defined using an acceptor-donor distance of 0.30 nm and an angle above 120 degrees, following commonly used hydrogen-bond distances in the literature.

During the MD simulation, a total of 4 hydrogen bonds were observed between 03 and the protein, while a total of 3 hydrogen bonds were observed between 29, the standard, and the protein. The graph clearly demonstrates that complex-03 has a higher number of hydrogen bonds throughout the entire simulation, whereas the standard has the lowest number of hydrogen bonds. The strength of binding is directly connected to the higher occurrence and longer duration of hydrogen bonds. Furthermore, by employing hydrogen-bond occupancy, crucial residues implicated in the formation of hydrogen-bonds for ligand recognition could be identified. The investigation of the relative frequencies of established ligand–protein hydrogen bond interactions was facilitated by the VMD “Hydrogen bonds” instrument. Ligand-03 preserves all the hydrogen bonds that were identified in the docked complex, but ligand-29 fails to preserve all of the hydrogen bonds discovered in the docked complex. In the case of the standard compound, it maintained interactions only with THR-207, ASP-210, AND VAL-206.

#### 3.7.5 Principal component analysis (PCA)

PCA is a nonparametric analysis, which serves as a dimensionality reduction technique. Generally utilized to investigate high-dimensional data from MD simulations into an informative low-dimensional space (52, 53). The collective motion’s complexity, which is linked to the stability of the system and the functions of proteins, could be investigated by adjusting different parameters and then simplifying the motion. Also, it can be utilized to characterize the many conformational variations that are associated with the process of protein folding as well as the open-close mechanism of ion channels (54).

**Figure 9:**
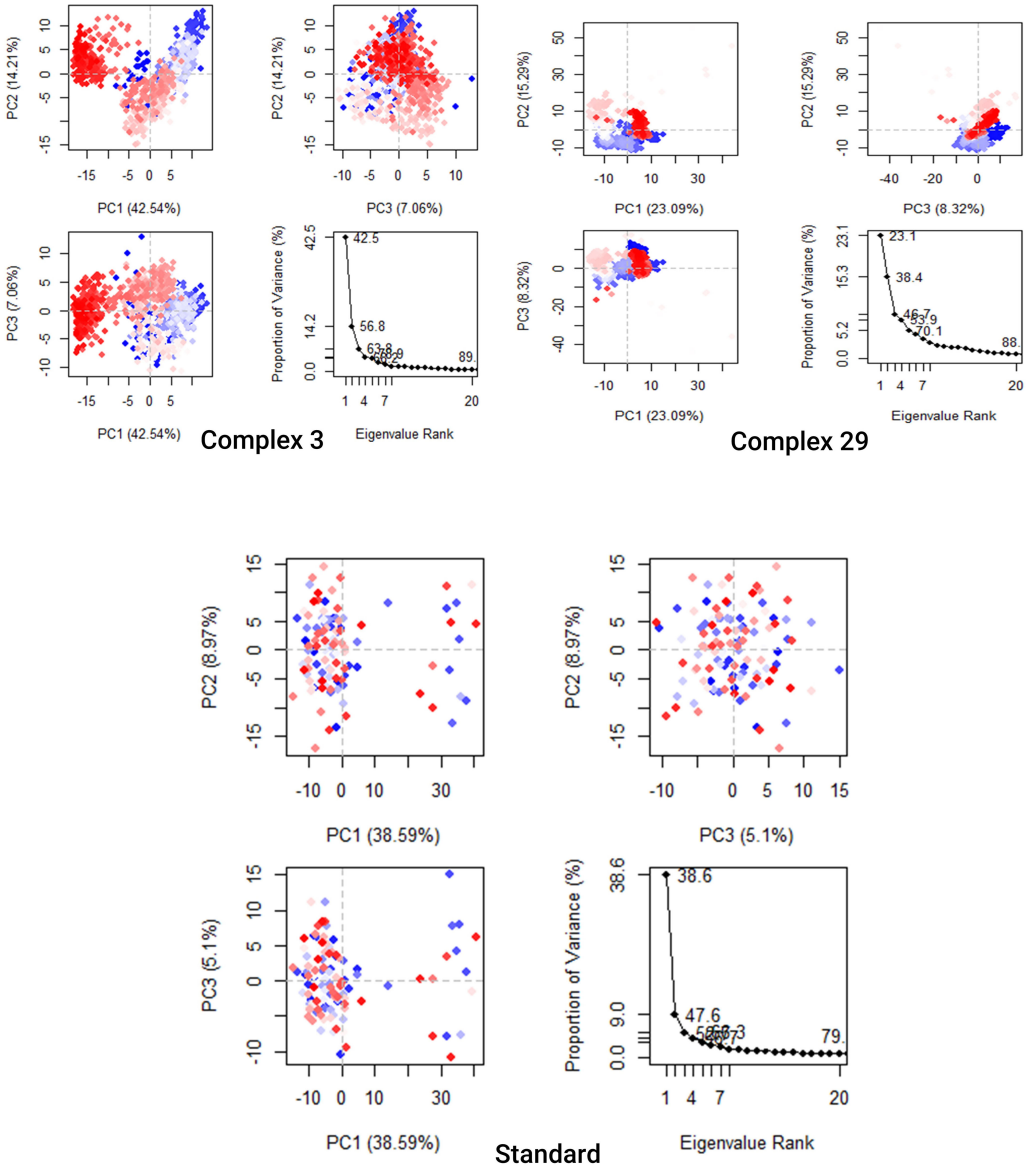
Principal Component Analysis Backbone of complex 03, 29, and Standard. Every data point represents the protein’s conformation about the X and Y axes. The simulation used a chromatic arrangement of blue and red dots to depict the extent of conformational alterations. The color gradient, ranging from blue to white to red, functioned as a visual depiction of the simulation’s duration. The color blue represents the start time step, the color white represents the intermediate time step, and the color red represents the final time step.

The analysis presented in **Figure 9** demonstrates that the top 20 PCs of the Ligand-03,29 system and the standard system contributed to 89%, 88%, and 79% of the overall variance, respectively. This indicates that the Ligand-29 and standard systems have a more restricted phase space and less performance flexibility compared to the Ligand-03 system.

The analysis presented in **Figure 9** demonstrates that the top 20 PCs of the Ligand-03,29 system and the standard system contributed to 89%, 88%, and 79% of the overall variance, respectively. This indicates that the Ligand-29 and standard systems have a more restricted phase space and less performance flexibility compared to the Ligand-03 system. In comparison to the PCA plots of Ligand-03,29 and ligand-standard the PC1 cluster exhibited the greatest variability, accounting for 42.54%, 23.09%, and 38.9% of the variance, respectively. The PC2 cluster demonstrated 14.21%, 15.29%, and 8.97% variability, while the PC3 cluster exhibited minimal variability, accounting for only 5.1% of the variance for the Ligand standard. The low degree of variability exhibited by PC3 for the Ligand standard, when compared to the PC1 and PC2 clusters, suggests that the binding of the Ligand standard is highly stable, and the structure is compact.

#### 3.7.6 Dynamics cross-correlation matrices (DCCM) analysis

**Figure 10:**
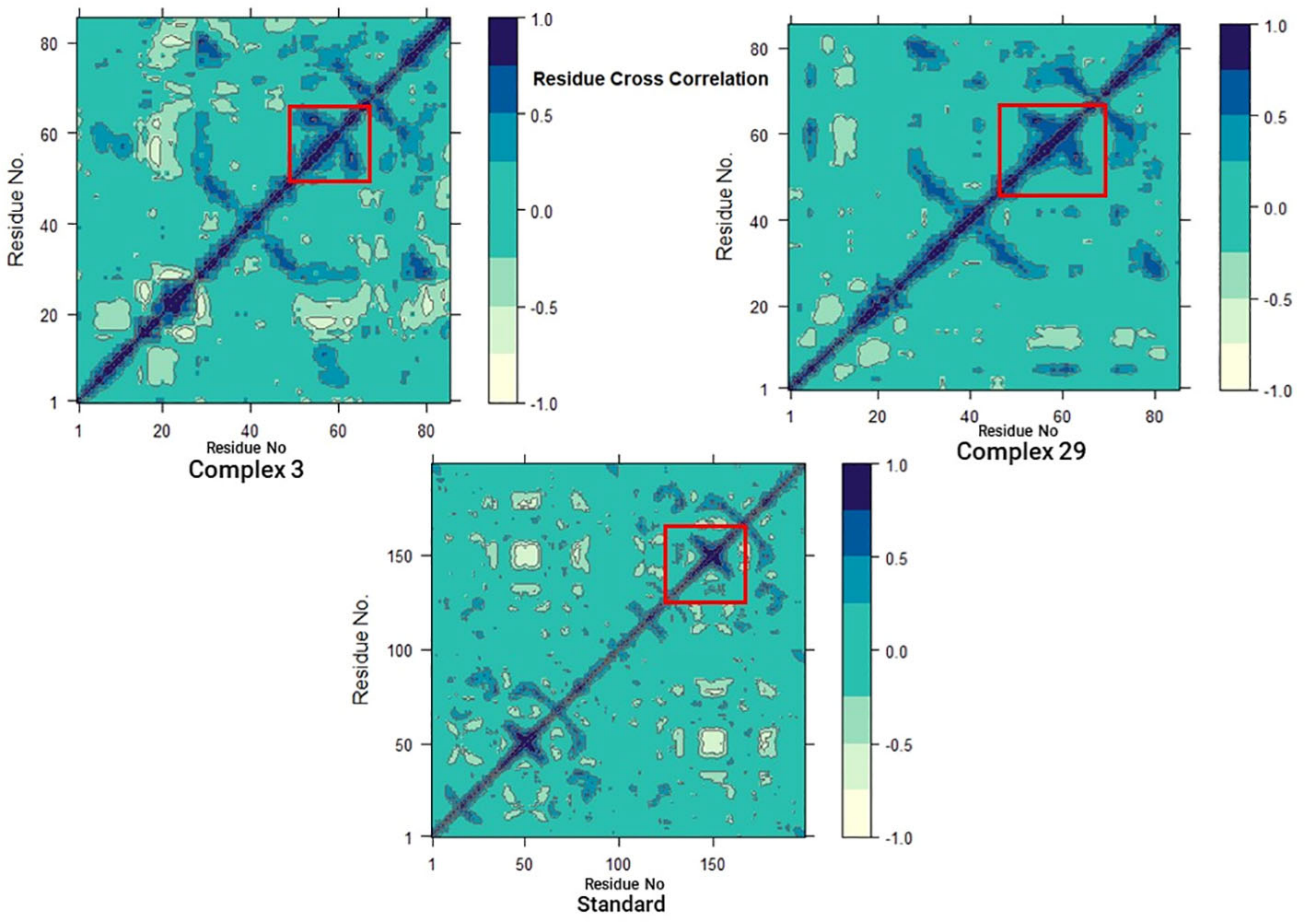
Dynamic cross-correlation matrix (DCCM) plots for (03, 29, and Standard).

In addition, to investigate the conformational changes of the targeted human armadillo domain of the APC (PDB ID: 3NMW) protein, all Cα atoms were analyzed using DCCM analysis (55, 56). 2D diagrams of the DCCM showed correlated motions between residues throughout the entire simulation process (**Figure 10**). DCCM showed an overall correlation ranging from -1.0 to 1.0 (light green to dark blue). Different colors were used to identify the different degrees of correlation between the residues and the darker the color, the stronger the correlation. Positive correlation (0 to 1) meant that the residues were moving in the same direction, while negative correlation (-1 to 0) meant that the residues were moving in the opposite direction.

A comparison of the DCCM diagrams of the three systems shows that the correlation behaviour of systems 03 and 29 in particular is significantly different from that of the standard system. Compared to the standard system, both systems exhibit a significant increase in positive correlation motions as seen in the black dashed boxes. Moreover, this increase is more pronounced in the 03 structure. This increase suggests that there are significant changes in protein-associated motions following ligand binding.

However, when the DCCM diagrams of ligand-03 and ligand-29 systems were analysed, it was revealed that the correlation movements differed significantly. In the ligand-29 system, the negative correlated motions were significantly reduced, while in the ligand-03 system, the overall positive correlated motions were relatively less changed. The reduction of negative correlated motions may help to stabilise the conformational state of the protein and indicate that it is more stable after ligand-29 binding.

#### 3.7.7 Binding free energy analysis

The Molecular Mechanics Poisson-Boltzmann Surface Area (MM-PBSA) method was used to calculate the binding free energy in protein-ligand complexes to analyze molecular binding interactions. This method considers various interactions, including binding and non-bonded forces like van der Waals and electrostatic forces. The binding free energy of these complexes was calculated using the MM-PBSA method, focusing on the last 20 nano-seconds of the trajectory. The binding free energy (ΔG bind) of ligands 03, 29, and the standard was determined using the MM-PBSA approach, which incorporates binding affinity scores. Lower values of ΔG indicate stronger binding affinities between proteins and ligands.

**Figure 11:**
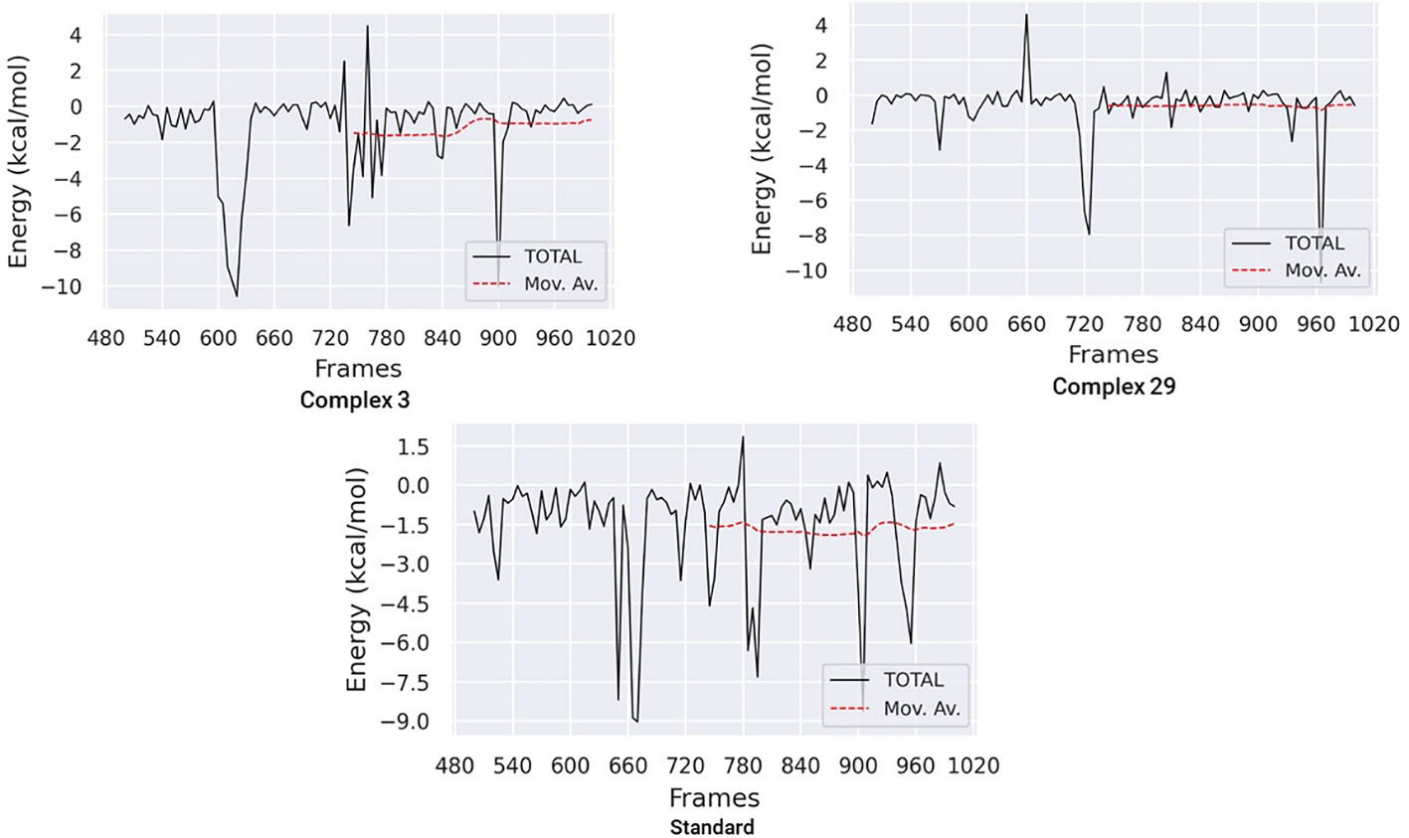
Binding free energy plots for ligand 03, ligand 29 and Standard.

**Table 6** and **Figure 11** demonstrate the correlation between the predicted free binding energies for ligands 03, 29 and the standard. The MM-PBSA study showed that the ligand 03 complex had higher binding energy and enhanced stability in comparison to ligand 29. The observations were confirmed by comparing molecular docking and MD simulation results using binding free energy calculations (**Table 6**).

**Table 6:** The findings of the ligands’ binding free energies.

Name	Delta;EVDW (kj/mol)	ΔEEEL (kj/mol)	ΔGPB (kj/mol)	ΔGNP (kj/mol)	ΔGDISP (kj/mol)	ΔG Binding free energy (kj/mol)
Complex-03	-1.57	-4.60	5.36	-0.30	0.00	-1.11
Complex-29	-0.80	-1.61	1.98	-0.15	0.00	-0.59
Standard	-1.89	-1.27	1.94	-0.31	0.00	-1.53

ΔGbind represents the total binding free energy.; ΔEVDW (kj/mol) represents the van der Waals contribution to the binding energy; ΔEEEL (kj/mol) represents electrostatic energy; ΔGPB (kj/mol) represents a polar portion of solvation; ΔGNP (kj/mol) represents non-polar part of solvation; ΔGDISP (kj/mol) represents dispersion.

## 4 Conclusion

In this study, we applied a wide range of computational approaches to identify the pharmacological effects of Resveratrol derivatives against CRC. These computational studies include molecular docking, theoretical ADMET, pharmacokinetics profile investigation, PASS prediction, and molecular dynamic simulations. Molecular docking analysis documented strong binding affinities to the target protein, compare to the standard drug capecitabine. In more precisely, ligands [(03) Resveratrol 3-beta-mono-D-glucoside, and (29) Resveratrol 3-Glucoside] has shown most favorable binding affinity. Subsequently, molecular dynamics simulations were performed to the top two compounds based on better binding energy, which confirm the stability of the drug-protein interactions, their stability. The MM-PBSA binding free energy analysis further supported the findings. Overall, these results highlight the potential of these molecules as effective candidates for the treatment of CRC.

## Data availability statement

The original contributions presented in the study are included in the article/**Supplementary material**. Further inquiries can be directed to the corresponding author.

## Author contributions

SA: Conceptualization, Formal analysis, Investigation, Methodology, Resources, Validation, Writing – original draft. MRI: Conceptualization, Data curation, Investigation, Methodology, Project administration, Visualization, Writing – original draft. AB: Conceptualization, Data curation, Investigation, Methodology, Project administration, Writing – original draft. MNI: Data curation, Investigation, Methodology, Project administration, Resources, Software, Writing – original draft. IB: Data curation, Formal analysis, Investigation, Methodology, Project administration, Resources, Visualization, Writing – review & editing. RS: Data curation, Formal analysis, Funding acquisition, Investigation, Methodology, Resources, Validation, Writing – review & editing. GA: Data curation, Formal analysis, Funding acquisition, Investigation, Validation, Writing – review & editing. MA-G: Data curation, Formal analysis, Funding acquisition, Investigation, Methodology, Writing – review & editing. MA-D: Formal analysis, Software, Supervision, Visualization, Writing – review & editing.
