# Supplementary material for: In silico evaluation of anti-colorectal cancer inhibitors by Resveratrol derivatives targeting Armadillo repeats domain of APC: molecular docking and molecular dynamics simulation
Source: Front Oncol. 2024 Apr 30;14:1360745. doi: 10.3389/fonc.2024.1360745 (PMC11091374; doi:10.3389/fonc.2024.1360745)

**Supplementary Table 1:** Biological Pass Prediction Spectrum Computation of Resveratrol Derivatives

| **No** | **PubChem ID** | **Antibacterial** | | **Antifungal** | | **Antineoplastic** | | **Antiparasitic** | |
| --- | --- | --- | --- | --- | --- | --- | --- | --- | --- |
|  |  | Pa | Pi | Pa | Pi | Pa | Pi | Pa | Pi |
|  | 1548910 | 0.306 | 0.058 | 0.431 | 0.043 | 0.626 | 0.040 | 0.423 | 0.026 |
|  | 445154 | 0.306 | 0.058 | 0.431 | 0.043 | 0.626 | 0.040 | 0.423 | 0.026 |
|  | 5281718 | 0.548 | 0.012 | 0.676 | 0.011 | 0.791 | 0.013 | 0.480 | 0.018 |
|  | 5962587 | 0.312 | 0.056 | 0.446 | 0.040 | 0.647 | 0.036 | 0.434 | 0.024 |
|  | 65522 | 0.186 | 0.131 | 0.201 | 0.136 | 0.461 | 0.083 | 0.297 | 0.057 |
|  | 185914 | 0.236 | 0.091 | 0.355 | 0.061 | 0.233 | 0.199 | 0.314 | 0.052 |
|  | 5281727 | 0.272 | 0.071 | 0.420 | 0.046 | 0.614 | 0.042 | 0.385 | 0.034 |
|  | 5388063 | 0.232 | 0.094 | 0.357 | 0.061 | 0.612 | 0.042 | 0.351 | 0.042 |
|  | 5318650 | 0.295 | 0.062 | 0.425 | 0.045 | 0.693 | 0.027 | 0.343 | 0.044 |
|  | 5473050 | 0.272 | 0.071 | 0.420 | 0.046 | 0.614 | 0.042 | 0.385 | 0.034 |
|  | 10178463 | 0.548 | 0.012 | 0.676 | 0.011 | 0.791 | 0.013 | 0.480 | 0.018 |
|  | 6255462 | 0.272 | 0.071 | 0.420 | 0.046 | 0.614 | 0.042 | 0.385 | 0.034 |
|  | 18475115 | 0.297 | 0.061 | 0.457 | 0.038 | 0.589 | 0.048 | 0.441 | 0.023 |
|  | 636928 | 0.388 | 0.033 | 0.547 | 0.024 | 0.683 | 0.029 | 0.565 | 0.010 |
|  | 11968839 | 0.548 | 0.012 | 0.676 | 0.011 | 0.791 | 0.013 | 0.480 | 0.018 |
|  | 45378267 | 0.440 | 0.023 | 0.636 | 0.015 | 0.738 | 0.020 | 0.667 | 0.007 |
|  | 10221222 | 0.355 | 0.042 | 0.430 | 0.044 | 0.743 | 0.019 | 0.355 | 0.041 |
|  | 3935161 | 0.312 | 0.056 | 0.446 | 0.040 | 0.647 | 0.036 | 0.434 | 0.024 |
|  | 6481476 | 0.353 | 0.043 | 0.501 | 0.030 | 0.642 | 0.037 | 0.464 | 0.019 |
|  | 135743666 | 0.331 | 0.049 | 0.349 | 0.063 | 0.308 | 0.148 | 0.439 | 0.023 |
|  | 44241616 | 0.204 | 0.113 | 0.463 | 0.037 | 0.280 | 0.165 | 0.387 | 0.034 |
|  | 11292556 | 0.547 | 0.012 | 0.664 | 0.012 | 0.833 | 0,008 | 0.380 | 0.035 |
|  | 10991144 | 0.309 | 0.057 | 0.375 | 0.056 | 0.432 | 0.093 | 0.222 | 0.087 |
|  | 53404893 | 0.353 | 0.043 | 0.501 | 0.030 | 0.642 | 0.037 | 0.464 | 0.019 |
|  | 46782864 | 0.306 | 0.058 | 0.431 | 0.043 | 0.626 | 0.040 | 0.423 | 0.026 |
|  | 54286634 | 0.548 | 0.012 | 0.676 | 0.011 | 0.791 | 0.013 | 0.480 | 0.018 |
|  | 25579167 | 0.548 | 0.012 | 0.676 | 0.011 | 0.791 | 0.013 | 0.480 | 0.018 |
|  | 53394008 | 0.579 | 0.010 | 0.686 | 0.010 | 0.701 | 0.026 | 0.490 | 0.017 |
|  | 73659198 | 0.236 | 0.091 | 0.299 | 0.081 | 0.469 | 0.081 | 0.148 | 0.130 |
|  | 53395097 | 0.330 | 0.049 | 0.433 | 0.043 | 0.694 | 0.027 | 0.328 | 0.048 |
|  | 53394009 | 0.579 | 0.010 | 0.686 | 0.010 | 0.701 | 0.026 | 0.490 | 0.017 |
|  | 129696259 | 0.306 | 0.058 | 0.431 | 0.043 | 0.626 | 0.040 | 0.423 | 0.026 |
|  | 129865704 | 0.343 | 0.045 | 0.259 | 0.102 | 0.482 | 0.077 | 0.174 | 0.109 |
|  | 72273590 | 0.283 | 0.067 | 0.366 | 0.058 | 0.565 | 0.053 | 0.381 | 0.035 |
|  | 77134557 | 0.331 | 0.049 | 0.362 | 0.059 | 0.451 | 0.086 | 0.448 | 0.022 |
|  | 129880647 | 0.448 | 0.022 | 0.655 | 0.013 | 0.695 | 0.027 | 0.346 | 0.043 |
|  | 129846314 | 0.301 | 0.060 | 0.421 | 0.046 | 0.775 | 0.015 | 0.363 | 0.039 |
|  | 76373349 | 0.309 | 0.057 | 0.378 | 0.055 | 0.697 | 0.027 | 0.341 | 0.045 |
|  | 54049345 | 0.172 | 0.144 | 0.277 | 0.092 | 0.586 | 0.048 | 0.302 | 0.055 |
|  | 69245694 | 0.548 | 0.012 | 0.676 | 0.011 | 0.791 | 0.013 | 0.480 | 0.018 |
|  | 129718809 | 0.275 | 0.070 | 0.419 | 0.046 | 0.717 | 0.023 | 0.280 | 0.063 |
|  | 59638843 | 0.579 | 0.010 | 0.686 | 0.010 | 0.701 | 0.026 | 0.490 | 0.017 |
|  | 92043645 | 0.579 | 0.010 | 0.686 | 0.010 | 0.701 | 0.026 | 0.490 | 0.017 |
|  | 129891988 | 0.309 | 0.057 | 0.442 | 0.041 | 0.278 | 0.167 | 0.333 | 0.047 |
|  | 139953844 | 0.283 | 0.067 | 0.366 | 0.058 | 0.565 | 0.053 | 0.381 | 0.035 |
|  | 45027804 | 0.565 | 0.011 | 0.664 | 0.012 | 0.771 | 0.015 | 0.391 | 0.033 |
|  | 134163433 | 0.297 | 0.061 | 0.508 | 0.029 | 0.580 | 0.049 | 0.281 | 0.062 |
|  | 57369399 | 0.232 | 0.094 | 0.357 | 0.061 | 0.612 | 0.042 | 0.351 | 0.042 |
|  | 71752002 | 0.579 | 0.010 | 0.686 | 0.010 | 0.701 | 0.026 | 0.490 | 0.017 |
|  | 71752014 | 0.312 | 0.056 | 0.446 | 0.040 | 0.647 | 0.036 | 0.434 | 0.024 |

**Supplementary Figure 1:** HOMO and LUMO surfaces of the ground state of the compounds, obtained by using the DFT/B3LYP/6-31G method.


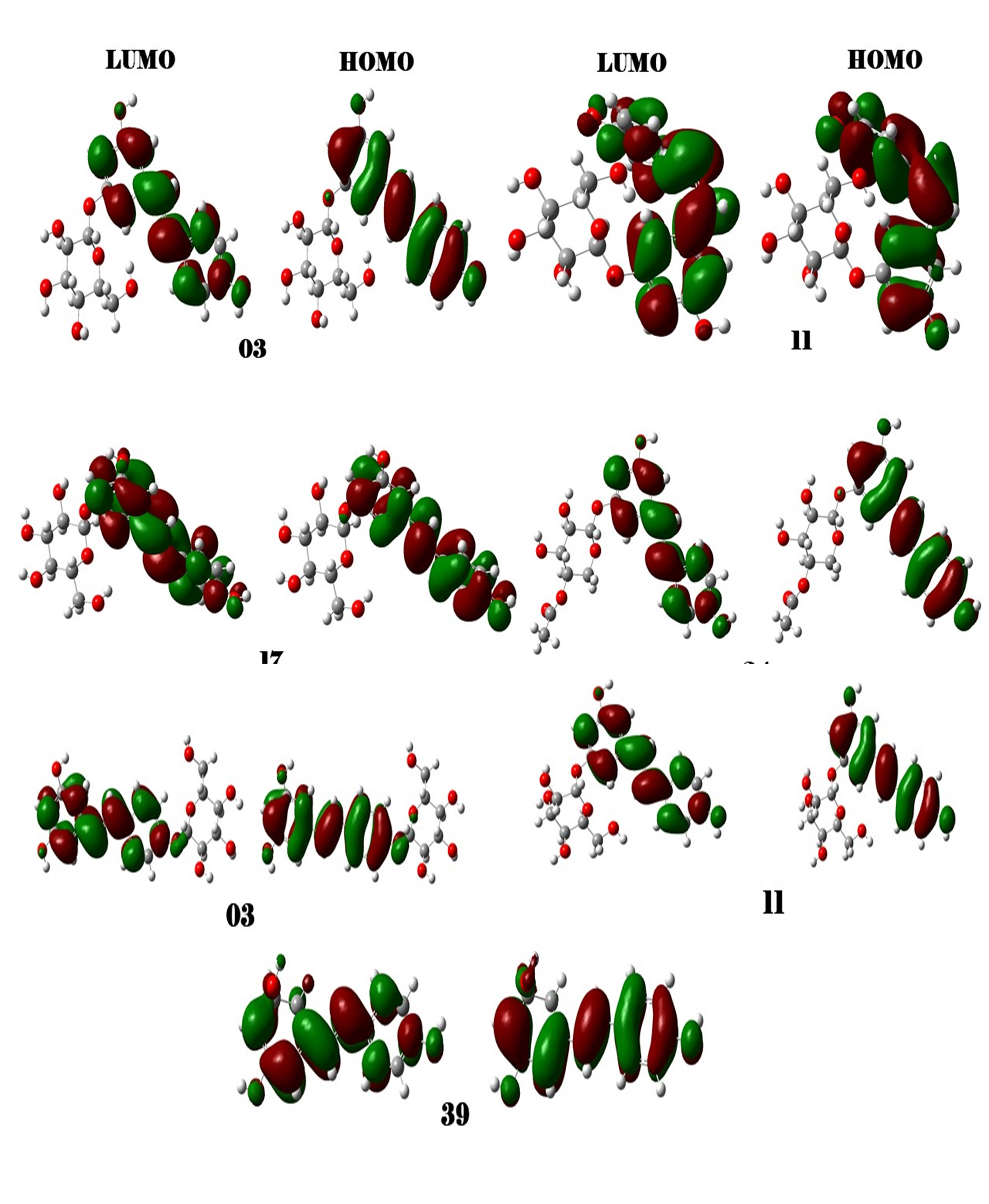

Supplement: Supplementary file 1 [file DataSheet_1.docx]
